# Enriching semantic knowledge bases for opinion mining in big data applications

**DOI:** 10.1016/j.knosys.2014.04.039

**Published:** 2014-10

**Authors:** A. Weichselbraun, S. Gindl, A. Scharl

**Affiliations:** a Faculty of Information Science, University of Applied Sciences Chur, Pulvermühlestrasse 57, CH-7004 Chur, Switzerland; b Department of New Media Technology, MODUL University Vienna, Am Kahlenberg 1, 1190 Vienna, Austria

**Keywords:** Web intelligence, Social Web, Big data, Knowledge extraction, Opinion mining, Sentiment analysis, Disambiguation, Contextualization, Common-sense knowledge, Concept grounding

## Abstract

This paper presents a novel method for contextualizing and enriching large semantic knowledge bases for opinion mining with a focus on Web intelligence platforms and other high-throughput big data applications. The method is not only applicable to traditional sentiment lexicons, but also to more comprehensive, multi-dimensional affective resources such as SenticNet. It comprises the following steps: (i) identify ambiguous sentiment terms, (ii) provide context information extracted from a domain-specific training corpus, and (iii) ground this contextual information to structured background knowledge sources such as ConceptNet and WordNet. A quantitative evaluation shows a significant improvement when using an enriched version of SenticNet for polarity classification. Crowdsourced gold standard data in conjunction with a qualitative evaluation sheds light on the strengths and weaknesses of the concept grounding, and on the quality of the enrichment process.

## 1 Introduction

Communication experts and decision makers aim to understand how stakeholders perceive their announcements and actions, and how news coverage and social media channels affect these perceptions. To address these questions, this article describes the integration and automated extension of semantic knowledge repositories. Building upon a novel approach to contextualized sentiment analysis [19], we introduce methods that can ground and enrich identified concepts. This integration of semantic knowledge repositories is an important stepping stone towards making sense of big data. Extracting factual and affective knowledge from these repositories will provide a deeper understanding of opinions expressed in user-generated content from social media platforms, news articles, scientific publications, etc.

**Fig. 1:**
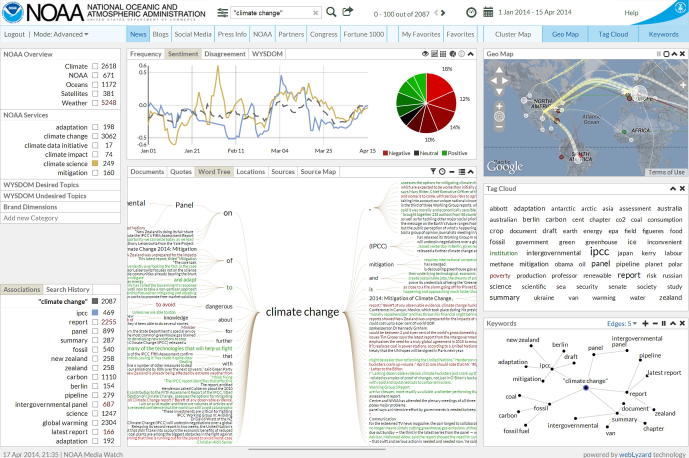
Screenshot of a Web intelligence portal built for the NOAA Climate Program Office, showing results for a query on “climate change” based on news media coverage between January and April 2014.

Knowledge extraction tools to analyze the Social Web typically provide frequency and sentiment metrics on either a document or sentence level. Sentiment is an important and insightful indicator. Even when measured accurately, however, this single metric often cannot address fundamental questions posed by decision makers. Communication experts who are responsible for marketing and public outreach campaigns, for example, want to know if their message reaches intended groups, how their communication strategy impacts observable patterns in online coverage, and which portion of the identified sentiment actually refers to their organization. The U.S. National Oceanic and Atmospheric Administration (NOAA) is a good example. The NOAA Climate Program Office has adopted the authors’ previous work on opinion mining as an essential part of its online evaluation strategy. Fig. 1 shows a screenshot of the system, which is based on the webLyzard big data and Web intelligence platform (www.weblyzard.com). The dashboard uses color coding to embed sentiment information into various interface components including a relevance-ranked list of search results, trend charts, and a portfolio of other interactive visualizations such as tag clouds, keyword graphs, word trees and geographic maps [15]. Communication experts at NOAA use the system to track whether social media users associate NOAA with “climate change”, for example, which is an important aspect of their communication and outreach goals. With regard to sentiment analysis, this poses an interesting challenge because the term “climate change” typically carries a negative connotation. In such cases, it is imperative to differentiate the sentiment of concepts that are merely co-referenced in a document (“NOAA”, “climate change”), and opinions that are directed towards an organization.

User-generated product reviews are another example illustrating the importance of identifying specific opinion targets when analyzing the Social Web. Users tend to comment not only on a product in general (e.g., digital camera), but also on its various aspects – shutter speed, quality of the lens, retail price, etc. The observable preference of users to compare features across products rather than assessing them in isolation underscores the need for flexible approaches to concept grounding and enrichment, which are granular enough to distinguish between the specific aspects of an entity. For the evaluations in Section 4, we therefore use reviews from Amazon.com about electronics and software products as well as reviews from the Internet Movie Database (www.imdb.com) in the categories comedy, crime and drama.

## 2 Related work

Many opinion mining tools rely on sentiment lexicons as linguistic resources that attach polarity values and strengths to sentiment terms. Static polarity values may serve as a good baseline, but a closer examination reveals the need for more differentiated approaches. Cambria and White emphasize the need for a shift from simple syntactic (bag-of-words) approaches to semantic (bag-of-concept) or even pragmatic (bag-of-narratives) ones in their extensive review on natural language processing [5]. Depending on the context, a term might lose its opinionated characteristic, or its polarity might change – e.g., “good” expressing a positive emotion versus “good” as the cargo of a freight train.

Gangemi et al. [9] emphasize the importance of sentiment contextualization as one of seven major challenges in the area of opinion holder and target detection. Existing context-aware approaches use vector space modeling [6], invoke language models [11], or apply sentence- and discourse-based context shifters [20], rule-based approaches [7] or linguistic patterns [21]. Xia et al. [22] address the problem of contextual polarity change by employing an ensemble of part-of-speech (POS) features combined with a sample selector. The sample selector uses principal component analysis to select samples from the source domain that are similar to the target domain. Enriching sentiment lexicons with context knowledge is another research avenue being pursued [12]. Gindl et al. [10] separate ambiguous sentiment terms from terms with stable polarity, a process that yields contextualized sentiment lexicons. Embedding context information into the lexicon allows adapting an ambiguous term’s polarity if the context indicates a polarity shift.

Structured knowledge contained in external linguistic repositories can support this contextualization process. Efforts to extend the well-known WordNet repository [8] have resulted in language resources such as SentiWordNet [1] and WordNetAffect [17]. The former attaches objectivity and polarity values to WordNet synsets, while the latter enriches WordNet with labels for affective categories. Tsai et al. [18] present another approach to enriching language resources. They apply iterative regression and a random walk strategy to label ConceptNet [16] elements with sentiment values. Poria et al. [13] merge SenticNet [3] and WordNetAffect to provide emotive labels for SenticNet. SenticNet itself uses ConceptNet by blending its knowledge with WordNetAffect, and inferring concept polarities from the Hourglass of Emotion [2].

Building on previous research into cross-domain contextualization [10] and complementing related work that applies common and common-sense knowledge to improve sentiment analysis [4], this paper specifically targets the problem of correctly interpreting ambiguous sentiment terms. We ground such terms depending on their actual usage to unambiguous concepts in knowledge sources such as ConceptNet and WordNet. In contrast to Poria et al. [13], our approach is based on bi-polar sentiment values rather than emotive categories. The identification and contextualization of ambiguous sentiment terms [10] is applied to SenticNet 3, using ConceptNet 5.1 to further enrich contextualized lexicons with concept knowledge.

## 3 Method

SenticNet [3] is a lexical resource that provides polarity and sentic information grouped into four categories: attention, pleasantness, sensitivity, and aptitude. As an extensive knowledge base applicable across domains, it would benefit from means to handle the context of polysemous sentiment terms. Currently, SenticNet assigns one polarity value to each sentiment term. A query for the term *approach*, for instance, yields positive polarity in conjunction with a neutral value for pleasantness, positive values for attention and aptitude, and a negative value for sensitivity. SenticNet does not indicate that the term *approach* refers to multiple concepts (i.e., senses). The concept of *approach* referring to *an action intended to deal with a problem or situation*, for example, has a different sentiment compared to occurrences that indicate *movement*.

This section summarizes the sequence of steps to contextualize SenticNet terms and ground them to common-sense and common knowledge, i.e. ConceptNet nodes and WordNet senses. The gained knowledge is used to enrich SenticNet concepts with context information from WordNet definitions and ConceptNet statements. This increases the coverage of SenticNet and paves the way for correctly interpreting ambiguous sentiment terms.

**Fig. 2:**
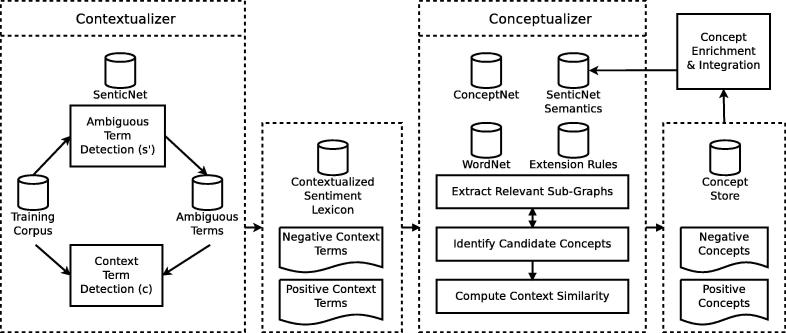
Overview of the contextualization, concept grounding and enrichment framework.

Fig. 2 illustrates the approach. SenticNet is transformed into a sentiment lexicon (Section 3.1), pre-processed and contextualized using a domain-specific training corpus. The Contextualizer component (Section 3.2) uses this lexicon to identify ambiguous sentiment terms based on their statistical properties in the training corpus. A Naïve Bayes approach then extracts positive and negative context terms that describe the use of the ambiguous term in a specific context.

To ground these ambiguous terms, we use two similarity measures: vector space similarity for ConceptNet (Section 3.3), and graph-based similarity for WordNet [19]. Both Conceptualizers (i) extend context terms with SenticNet semantics, (ii) query structured knowledge sources such as ConceptNet and WordNet, (iii) apply constraints stored in a database of semantic background knowledge to identify positive and negative candidate concepts for grounding the ambiguous term, (iv) semantically enrich the context terms and candidate concepts to compute their similarity, and (v) ground the ambiguous terms to the concepts that best describe their context. The ambiguous term “approach”, for example, gets grounded to the positive concept “movement (change in position)” and the negative concept “approach (deal with a problem or situation)”.

The enrichment process outlined in Section 3.4 draws upon the derived groundings to further extend the semantic description of the ambiguous terms with context information and concept groundings, and to integrate them with (or link them to) relevant data from the original knowledge sources.

### 3.1 Transforming SenticNet into a sentiment lexicon

SenticNet contains polarity, semantics and sentics for more than 14,000 terms – including stop words and terms of low polarity strength that are of limited value in sentiment analysis. To overcome this *insignificant low-value problem* [18], we remove stop words and inflate the strengths of weakly polar terms to the binary values 1 and −1, increasing their impact. At the same time, we reduce the weights of very weak terms to zero. This prevents them from polluting the keyword look-up, while still retaining them in case the system later identifies them as ambiguous terms in the contextualization process.

### 3.2 Contextualization

Contextualization identifies ambiguous terms and adds context information for their disambiguation to a sentiment lexicon [10]. We define context as the set of terms that co-occur with ambiguous terms. For each ambiguous term, the lexicon stores the co-occurring terms together with their probability to accompany the ambiguous term in either a positive or negative context as determined by the training corpus. Once properly trained, the contextualized lexicon delivers context-specific sentiment values for ambiguous terms. This contextualization can be achieved through the following processing steps:

1. **Identify ambiguous sentiment terms** based on their frequency distribution in the positive and negative sub-collections of the training corpus.
2. **Integrate context terms** by analyzing the co-occurrence of ambiguous terms to estimate the probability of a positive/negative context given a specific pair of ambiguous term and context term.
3. **Improve document classification** by identifying context terms that indicate whether the ambiguous term is used positively or negatively. After the disambiguation step, the resulting polarity goes into a keyword look-up algorithm using rule-based negation detection.

### 3.3 Concept grounding with ConceptNet

The Conceptualizer performs a series of processing steps to ground ambiguous sentiment terms to ConceptNet nodes and WordNet concepts: (i) extract positive and negative context terms from the contextualized sentiment lexicon; (ii) query WordNet and ConceptNet for relevant sub-graphs describing the ambiguous term, its context terms, as well as candidate concepts and their respective context terms; and (iii) compute the similarity between the sub-graphs retrieved for the ambiguous term and the sub-graphs extracted for the candidate concept to determine the final concept grounding.

The following sections describe the process for grounding ambiguous sentiment terms to ConceptNet. Please refer to Weichselbraun et al. [19] for a detailed discussion of the WordNet-based grounding algorithm.

#### 3.3.1 Extraction of context terms

**Fig. 3:**
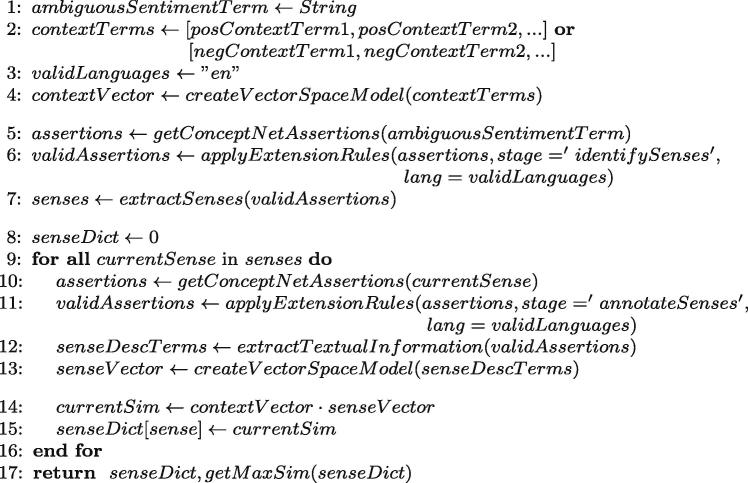
Computation of (i) ConceptNet candidate concepts and their similarity to the positive or negative interpretation of the ambiguous sentiment term and (ii) the maximum similarity score ${\mathrm{sim}}_{C_{\pm }\text{,}\max}$.

For each ambiguous sentiment term, the contextualized lexicon contains context terms and their conditional probabilities (Fig. 3, lines 1–2). Occurrences of *approach* in the Amazon electronics corpus, for example, yield context terms related to software products and their usability: *Armaan* $\left(p_{\mathrm{pos}}=0.06\right)$, *Kaspersky* $\left(p_{\mathrm{pos}}=0.10\right)$, *India* $\left(p_{\mathrm{pos}}=0.11\right)$, *exercises* $\left(p_{\mathrm{pos}}=0.89\right)$, *licence* ($p_{\mathrm{pos}}=0.89$), *screen* $\left(p_{\mathrm{pos}}=0.89\right)$.

We discard context terms with conditional probabilities between 0.4 and 0.6 and include the sentiment term’s SenticNet semantics into the context term list. The probability $\left(p_{\mathrm{pos}}\right)$ of those terms has a default of 0.99 if related to positive context terms, and 0.01 if they are related to negative ones.

#### 3.3.2 Identification of candidate concepts

The Conceptualizer extracts relevant sub-graphs from structured knowledge sources, which provide candidate concepts for the concept grounding (Fig. 3, lines 3–7). Input filters remove irrelevant or misleading information based on hand-crafted extension rules that define relevant languages and relation types for each step. For example, the algorithm only uses hypernym, hyponym, instance and synonym relations to determine potential candidate senses for the concept grounding (Fig. 3, line 6), but draws upon the full set of ConceptNet relations for obtaining background information on these candidate concepts (Fig. 3, line 11). For scalability reasons, the algorithm limits the size of the extracted sub-graph after filtering to 5000 assertions per node.

A ConceptNet query for *approach* yields a sub-graph with a total of 890 assertions. Based on the extension rules, we limit this result to English-speaking assertions that indicate a hypernym, hyponym, instance or synonym of the input term, obtaining a significantly smaller sub-graph. The Conceptualizer extracts the concepts participating in these assertions, which returns a total of 32 candidate concepts to ground the ambiguous sentiment term *approach*.

In a next step, we retrieve assertions for every candidate concept from ConceptNet. The following filtering step allows a much richer set of relations such as PartOf, SubjectOf, HasProperty, etc. Table 1 shows a selection of identified candidate concepts and the number of corresponding assertions. ConceptNet contains (i) qualified entries that include annotations such as the part of speech and term usage and (ii) unqualified entries that lack this information and roughly correspond to plain SenticNet entries. Since unqualified entries are more general, they yield a much larger number of context assertions.

**Table 1:** Candidate concept selection and extracted textual information for the term *approach*.

Concept · assertions	Retrieved context terms
1. Come · 11,990	Come, toward, something, move, …
2. Approach/v (move towards) · 54	Approach, move, towards, draw, drive, …
3. Movement/n (a natural event that involves a change in the position or location of something) · 25	Movement, that, location, change, event, something, position, involve, natural, …
⋯	⋯
32. Approach/n (ideas or actions intended to deal with a problem) · 3	Idea, deal, approach, with, intend, action, situation, problem, …

#### 3.3.3 Similarity computation and grounding

The Conceptualizer then extracts textual information from the candidate concepts and assertions, and transforms this information into a vector space representation $\left(\overset{\to }{V_{i}}\right)$. Table 1 illustrates this process with a selection of the candidate concepts obtained for the term *approach*, and the respective textual information that is used to create the vector space representation.

We then use the cosine similarity measure to compute the similarity between a concept’s ($C_{i}$) vector space representation ($\overset{\to }{V_{i}}$) and the vector space representation of the positive ($\overset{\to }{V^{+}}$) and negative context terms ($\overset{\to }{V^{-}}$) from the preceding extraction step (Section 3.3.1).

(1) $${\mathrm{sim}}_{C_{i}+}=\frac{\overset{\to }{V_{i}}\cdot \overset{\to }{V^{+}}}{\left|V_{i}|\cdot |V^{+}\right|}$$

(2) $${\mathrm{sim}}_{C_{i}-}=\frac{\overset{\to }{V_{i}}\cdot \overset{\to }{V^{-}}}{\left|V_{i}|\cdot |V^{-}\right|}$$

To ground the positive meaning of the ambiguous concept, the Conceptualizer first determines the similarity score (${\mathrm{sim}}_{C_{i}+}$) for every concept $C_{i}$ and its maximum ${\mathrm{sim}}_{C_{+}\text{,}\max}$ (Fig. 3). It then assembles a list of candidate concepts $C^{+}$ with a minimum similarity ${\mathrm{sim}}_{C_{j}+}\cdot f>{\mathrm{sim}}_{C_{+}\text{,}\max}$. Experiments have shown that a factor of f=3 yields good results. In the next step, the Conceptualizer computes the corresponding concept list for the term’s negative meaning $C^{-}$. If ambiguous concepts such as */c/en/work* or */c/en/music* occur in both lists, the Conceptualizer removes them and returns the remaining three concepts with the highest similarity score ${\mathrm{sim}}_{C_{j}+}$ for the positive and ${\mathrm{sim}}_{C_{j}-}$ for the negative grounding.

### 3.4 Enrichment

The enrichment process adds positive and negative context terms to extend the expressiveness of semantic knowledge bases such as SenticNet. This helps adapt them to a specific domain by using an appropriate training corpus in the contextualization. The ConceptNet Conceptualizer returns positive and negative concepts. In addition, we annotate the positive and negative usage of the ambiguous term with their WordNet senses and retrieve the corresponding definitions together with WordNet synonyms and antonyms.

## 4 Evaluation

Humans excel at interpreting contradictory and context-dependent evidence. Leveraging this ability, the evaluation of the presented approach has been conducted as part of the uComp project (www.ucomp.eu), which merges collective human intelligence and automated knowledge extraction methods in a symbiotic fashion. A quantitative evaluation of context-aware sentiment analysis is followed by a hybrid assessment of the concept grounding and concept enrichment processes, thus combining a qualitative approach with quantitative measures obtained through the Crowdflower marketplace (www.crowdflower.org).

We modeled the evaluation of the Contextualizer as a polarity classification task. Reviews from Amazon.com and IMDb.com provided the labeled data. The rating categories served as polarity classes. For Amazon reviews, ratings of one and two were labeled as “negative”, and ratings four and five as “positive”. IMDb reviews range from one to ten stars. To standardize these ratings, we treated one and two IMDb stars as equivalent to a rating of one on Amazon, three and four stars as a rating of two, and so forth. An Amazon rating of three and an IMDb rating of five or six was considered neutral and therefore disregarded.

We applied 10-fold cross-validation to the five corpora. Table 2 compares the size of these corpora based on the word count and number of sentences.

**Table 2:** Amazon and IMDb corpus characteristics.

Corpus	Reviews	Total counts		Avg per review	
		Sent.	Words	Sent.	Words
Amazon electronics	2000	19,911	298,622	10	149
Amazon software	2000	24,120	380,760	12	190
IMDb comedy	2000	25,481	410,874	13	205
IMDb crime	2000	30,155	494,686	15	247
IMDb drama	2000	27,026	432,820	14	216

### 4.1 Quantitative evaluation – sentiment analysis

The quantitative evaluation compares context-aware sentiment analysis with a context-unaware baseline. The recall, precision, and *f*-measure values of Table 3 show significant improvements (↑), significant declines (↓) and insignificant changes (·) based on Wilcoxon’s rank sum test at the 0.05 level.

**Table 3:** 10-fold cross validation of the baseline (b) versus context-aware (c) sentiment analysis.

*Corpus*			$R_{b}$	$R_{c}$	$p_{R}$	$P_{b}$	$P_{c}$	$p_{P}$	$F_{b}$	$F_{c}$	$p_{F}$	$A_{b}$	$A_{c}$	$p_{A}$
*Amazon reviews*														
Electronics		+	0.62	0.66	↑	0.83	0.83	·	0.71	0.74	↑	0.65	0.70	↑
		−	0.74	0.77	·	0.48	0.58	↑	0.58	0.66	↑			
Software		+	0.60	0.60	·	0.82	0.91	↑	0.69	0.72	↑	0.63	0.65	·
		−	0.71	0.81	↑	0.44	0.40	·	0.54	0.53	·			

*IMDb reviews*														
Comedy		+	0.59	0.80	↑	0.92	0.89	↓	0.72	0.84	↑	0.64	0.83	↑
		−	0.82	0.87	↑	0.36	0.77	↑	0.50	0.82	↑			
Crime		+	0.60	0.95	↑	0.80	0.49	↓	0.68	0.64	↓	0.63	0.73	↑
		−	0.69	0.66	↑	0.46	0.97	↑	0.55	0.78	↑			
Drama		+	0.57	0.73	↑	0.86	0.93	↑	0.69	0.82	↑	0.61	0.79	↑
		−	0.72	0.90	↑	0.36	0.66	↑	0.48	0.76	↑			

For electronics, the Contextualizer significantly improved accuracy from 65% to 70% (the improvement from 63% to 65% in the software category was not significant). The three IMDb datasets on comedy, crime and drama also showed significant improvements from 64% to 83%, 63% to 73% and 61% to 79%, respectively. The contextualization had a positive effect on recall and precision, with significant increases across all corpora except for precision in positive comedy reviews (decline from 92% to 89%), and precision and *f*-measure in positive crime reviews (decline from 80% to 49% and 68% to 64%, respectively).

### 4.2 Qualitative evaluation – grounding

This section presents a qualitative evaluation of the concept grounding and concept enrichment processes. Table 4 contains context terms extracted from the Amazon product (electronics, software) and the IMDb movie datasets (comedy, crime, drama). An analysis of context terms shows their connection to particular domains. The most significant context terms from Amazon for the ambiguous term *development*, for example, contain the names of products (Dreamweaver, PaperPort and Windows Live OneCare) and companies (NDUC = Nova Development User Community). IMDb typically yields the names of actors, producers and fictive characters (Chadwick, Chazz, Redford, etc.), or more generic results for ambiguous terms such as *challenge* (role, student).

**Table 4:** Selected ambiguous terms, their respective context terms, and the corresponding ConceptNet and WordNet grounding.

Term		Context term	ConceptNet	WordNet
*Amazon reviews*				
Adventure	(+)	During nostradamus diary	Activity, magical journey, fun trip	Wild and exciting undertaking
	(−)	Educational windvd frame	Software, band, video game	Wild and exciting undertaking
Development	(+)	Creating dreamweaver nduc	Progression from simpler to more complex forms	Growth
	(−)	Onecare paperport auction	Recent event that has some relevance for the present situation	Development
God	(+)	Reading cuppa hdd	One of greater rank or station or quality	Deity
	(−)	Folder quicklaunch netbook	An incorporal being believed to have powers to affect the course of human events	God

*IMDb reviews*				
Challenge	(+)	Maris hal role	Confrontation (call into challenge)	A call to engage in a contest or fight
	(−)	Skulls luke student	Invite, call into question	A call to engage in a contest or fight
Ridiculous	(+)	Chazz jimmy chadwick	Funny	Farcical
	(−)	Tremors burt shortbus	Goofy	Absurd
Plot	(+)	Brita ryan jai	Piece of fiction that narrates a chain of related events	Chart or map showing the movements or progress of an object
	(−)	Hancock redford surratt	Conspiracy	Chart or map showing the movements or progress of an object

Table 4 also contains the ConceptNet grounding that uses up to 100 context terms to assign positive and negative concepts to each ambiguous term. This grounding performs remarkably well, especially considering that the algorithm – depending on the ambiguity of the concept – often has to select suitable concepts out of hundreds of candidate terms. The system distinguishes real-world *adventures* (activities, trips, journeys) from virtual ones (software, video games), for example, and even recognizes subtle nuances such as *follow a guidance* versus *consequences that follow from an action*, or the difference between ridiculously *funny* and *goofy* movies.

Some of the included concepts such as */c/en/net/n* for *development* and the very general */c/en/write*_*work* concept for the ambiguous term *god* are less intuitive. This reflects the similarity metric’s slight bias towards concepts with more elaborate descriptions. Future work will address this issue by adding further pre-processing and normalization steps when extracting textual information from ConceptNet sub-graphs.

Since ConceptNet blends multiple knowledge sources, we also obtained statistics on the distribution of these sources among the grounded concepts. The concepts were grounded to terms originating from WordNet (45%), the Open Mind Common Sense project (27%; commons.media.mit.edu), and the online game Verbosity (10%; (www.gwap.com). The remaining concepts refer to other sources such as DBpedia.org and Wiktionary.org.

The right-hand column of Table 4 summarizes the results of the WordNet-based grounding. Compared to ConceptNet, this task seems less challenging due to the lower number of candidate concepts – e.g. WordNet only distinguishes between the sense *adventure.n.01* referring to a “wild and exciting undertaking (not necessarily lawful)”, *gamble.v.01* referring to “taking a risk in the hope of a favorable outcome”, and *venture.v.03* referring to “put at risk”. The lack of the required concepts in WordNet, however, means that the term *adventure* could not be disambiguated, and that the system could not ground the positive and negative usage of the ambiguous terms *challenge* and *plot* to different concepts.

The grounding of WordNet for *god* contradicts the ConceptNet result. We ascribe this to the different number of semantic categories per source, and the differences in the chosen disambiguation techniques (structural versus textual information). Future work will focus on the automated detection and resolution of such conflicts by integrating and better aligning background knowledge from multiple sources. The grounding of the other ambiguous terms is well-aligned between ConceptNet and WordNet.

### 4.3 Crowdsourced evaluation – grounding

To extend the qualitative evaluation discussed in the previous section, we conducted a quantitative evaluation of the concept grounding by compiling a list of qualified ConceptNet groundings, i.e. those that included a part of speech tag and a short textual description of the ambiguous term’s interpretation.

The evaluation corpus contained 897 positive and 725 negative concept groundings for Amazon, as well as 1273 positive and 968 negative groundings for IMDb. Each grounding was inspected by three human participants, which assigned a sentiment value between 5 (very positive) and 1 (very negative) to each grounded concept, yielding a total of 11,589 assessments. The average rating variance amounted to 0.17 (0.22) for Amazon (IMDb) review data. This evaluation provided insights into the nature of the observed ambiguities and the grounding process. The evaluators perceived 79% (72%) of the concepts from Amazon (IMDb) as neutral. The evaluation presented in Section 4.1 shows that this does not reflect their actual use in the review corpus, which emphasizes how difficult it is for human evaluators to determine a concept’s polarity based on its definition alone.

For concepts considered polar in the Amazon (IMDb) dataset, the assessors agreed in 61% (68%) of cases. Restricted to positive groundings, this figure increases to 64% (71%). This positive bias can be explained by concepts such as *attack, coma, debt* and *opposition*, which can represent interesting elements of a movie plot. Similarly, *addictive* computer games simulating armed *conflicts* and *warfare* often receive five-star ratings.

### 4.4 Enrichment process

Table 5 summarizes the results of the domain adaptation and enrichment process. The contextualization yielded domain-specific positive and negative context terms, which can be used in context-aware sentiment analysis [19]. The method extracted approximately four times more context terms for ambiguous sentiment terms in IMDb reviews than for Amazon reviews. This is in line with expectations since IMDb reviews contain more ambiguous sentiment terms and often include plot elements, which provide the Contextualizer with a rich selection of potential context terms.

**Table 5:** Enrichment statistics.

	Amazon reviews	IMDb reviews
*Contextualization*		
Positive context terms	793,948	2,060,333
Negative context terms	549,120	2,608,472

*ConceptNet grounding*		
– Grounded concepts	1018	1637
– Positive	2287 (2141 unique)	3649 (3248 unique)
– Negative	2072 (1773 unique)	3437 (2633 unique)

*WordNet grounding*		
– Senses and definitions	519	857
– Synonyms	3015 (2072 unique)	5012 (3245 unique)
– Antonyms	108 (94 unique)	159 (138 unique)

The context terms aided in disambiguating 1339 (2369) out of 1366 (2417) sentiment terms considered ambiguous in the Amazon (IMDb) corpus. The Conceptualizer successfully grounded 1018 (74.5%) concepts of the Amazon corpus to ConceptNet nodes and 519 (38.0%) to WordNet senses. For the IMDb corpus, we were able to link 1637 (69.1%) concepts to ConceptNet and 857 (36.2%) to WordNet senses. These results show that the Conceptualizer is able to successfully leverage ConceptNet’s higher expressiveness in terms of concepts and assertions.

Table 5 also indicates how often the Conceptualizer was only able to ground a concept to either a positive or a negative ConceptNet node. These numbers underscore the previous conclusion that corpora with a richer selection of context terms will yield more groundings.

We used the grounded WordNet concepts to integrate WordNet senses and definitions as well as synonyms and antonyms into the knowledge base. Due to the high interconnectedness of ConceptNet, we did not include ConceptNet assertions into the refined knowledge base. It is more efficient to directly query a grounded concept on ConceptNet, rather than to replicate these data.

### 4.5 Discussion

Section 4.1 demonstrates that the contextualization of ambiguous sentiment terms significantly improves the performance of sentiment analysis methods. The necessary language resources are stored in contextualized sentiment lexicons – including ambiguous sentiment terms and context information to help interpret their usage in the current document. The high number of extracted context terms indicates that ambiguous sentiment terms occur in a variety of settings that influence their interpretations. This observation does not confirm whether these interpretations correspond to common knowledge and common-sense knowledge concepts, which could contribute to an automatic detection and resolution of ambiguities.

The qualitative evaluation of Section 4.2 and the grounding statistics of Section 4.4 indicate that many identified ambiguities correspond to tangible concepts found in common-sense or common knowledge sources. At the same time, the crowdsourcing experiment of Section 4.3 reminds us that grounding helps to disambiguate different concept meanings, but that the overall setting (i.e., whether the text contains plot elements or describes a user’s attitude towards software or computer games) also plays an important role in determining the correct interpretation of contextualized knowledge resources.

Enriching semantic knowledge bases such as SenticNet with information on (i) potentially ambiguous sentiment terms, (ii) positive and negative context terms and (iii) the grounding of these interpretations to common-sense and common knowledge paves the way for adapting sentiment analysis components to address these ambiguities in a systematic manner. This approach capitalizes upon past efforts to create and refine such language resources.

As demonstrated by the experiments in Section 4.2, these resources will be domain-dependent unless generic contextualization methods [10] are deployed to remove domain-specific context terms from the contextualized sentiment lexicon prior to the contextualization step.

## 5 Conclusion and future work

This article introduces a novel method to extend sentiment lexicons with concept knowledge, which aims to increase the lexicons’ coverage and derive concept information for subsequent opinion mining. We use SenticNet terms and their polarity values to generate a baseline sentiment lexicon, identify ambiguous sentiment terms, and extract context information for disambiguating these terms in the application phase. Based on the extracted context information, the method grounds the ambiguous terms and then obtains conceptual knowledge from two different structured resources, WordNet and ConceptNet.

A quantitative analysis of the contextualization approach demonstrates the importance of context for correctly assessing a term’s polarity. The quantitative analysis draws upon a 10-fold cross-validation on corpora from five different domains – electronics and software product reviews from Amazon as well as reviews from the IMDb categories comedy, crime, and drama. A qualitative analysis shows that the presented ConceptNet grounding performs well and successfully grounds a considerable percentage of ambiguous concepts – 74.5% as compared to 38.0% achieved with a previous WordNet-based approach [19].

Leveraging the concept grounding to semantically enrich SenticNet improves its expressiveness and provides valuable background information for advanced sentiment analysis tasks such as opinion holder and target extraction [9]. The presented method expands SenticNet and considerably lowers the effort required to use it in conjunction with ConceptNet and WordNet.

Future work will use crowdsourcing to annotate large sentiment corpora on the sentence level. This will provide a gold standard to refine the contextualized lexicon – e.g., avoid the inclusion of context terms that do not co-occur with the ambiguous terms within the same sentence. We will also process larger corpora such as the knowledge archive of the Media Watch on Climate Change (www.ecoresearch.net/climate), a public Web portal using the webLyzard Web intelligence platform (www.weblyzard.com) to aggregate and analyze online coverage about climate change and related environmental issues. Its dashboard resembles the NOAA portal shown in Fig. 1, including a knowledge co-creation component [15] that tackles two areas where time- and resource-efficiency are of particular importance: synchronous collaboration and the analysis of big data from social sources [14]. When extending the presented approach, therefore, we will continue to place special emphasis on scalability and throughput. Large corpora will enable us to (i) include very specific terms and yield more generic contextualized lexicons, (ii) create a cross-domain version of the enriched SenticNet repository by using the technique introduced by Gindl et al. [10] to remove domain-specific terms from the contextualized sentiment lexicon, and (iii) infuse the contextualized lexicons with the retrieved concept knowledge.
